# Attacks With Urinary Incontinence Without Convulsions: Complete Atrioventricular Block Mimicking an Epileptic Seizure

**DOI:** 10.7759/cureus.49552

**Published:** 2023-11-28

**Authors:** Shuichiro Neshige, Narumi Ohno, Hirofumi Maruyama

**Affiliations:** 1 Clinical Neuroscience and Therapeutics, Graduate School of Biomedical and Health Sciences, Hiroshima University, Hiroshima, JPN

**Keywords:** loss of consciousness, convulsion, temporal love epilepsy, syncope, seizure

## Abstract

Differentiating between syncope and epileptic seizures can be challenging when a specific medical history is not available. We herein report a 70s man who exhibited recurrent, brief unresponsiveness while at rest on five occasions over a year. While there were no convulsions, the patient consistently reported urinary incontinence. These events were preceded by an epigastric rising sensation without chest symptoms, suggesting a possible diagnosis of temporal lobe epilepsy, and subsequent EEG revealed temporal semi-rhythmic delta activity. In contrast, the ECG revealed a left bundle branch block, while the initial Holter ECG showed no abnormalities. However, subsequent follow-up examinations revealed a complete atrioventricular block necessitating permanent pacemaker implantation. It is important to exercise caution in the interpretation of EEG findings. Moreover, instances of 'urinary incontinence without convulsion' may indicate non-epileptic events.

## Introduction

Differentiating between syncope and epileptic seizures can be straightforward when a clear and specific medical history is available. Thus, clinicians should thoroughly assess patients for indicators suggestive of either syncope or epileptic seizures. For instance, clinical signs like tongue biting, head version, cyanosis, and postictal drowsiness typically point toward epileptic seizures [1]. Conversely, findings indicative of syncope encompass symptoms like sweating, nausea, and pallor; however, nausea or abdominal discomfort might be a non-specific finding, as it may manifest in the context of epileptic seizures as an epigastric rising sensation [2,3]. We experienced a patient with an abdominal aura that manifested just before the onset of loss of consciousness. Additionally, initial screening using electroencephalography (EEG) and electrocardiography (ECG) yielded abnormal results, further complicating the diagnostic process. We herein present this case to emphasize the importance of avoiding hasty interpretations of EEG findings.

## Case presentation

A right-handed man in his 70s exhibited recurrent, brief unresponsiveness at rest on five occasions over a year. More specifically, while watching television with his wife, he described an abdominal aura characterized by an epigastric rising sensation lasting approximately 30 seconds without chest symptoms. This was followed by a loss of responsiveness with gaze fixation for 2 to 3 minutes. The presence or absence of automatisms was not available during the event. However, he never reported other epileptic auras, such as déjà vu, fear, or panic. There were no convulsions, but urinary incontinence was consistently reported. Within a few minutes, he regained full consciousness. All events occurred at rest. Specifically, they occurred while the patient was sitting or lying on their side. Thus, the presence or absence of generalized weakness was not available (the patient would lean against the backrest but never fell over).

**Figure 1:**
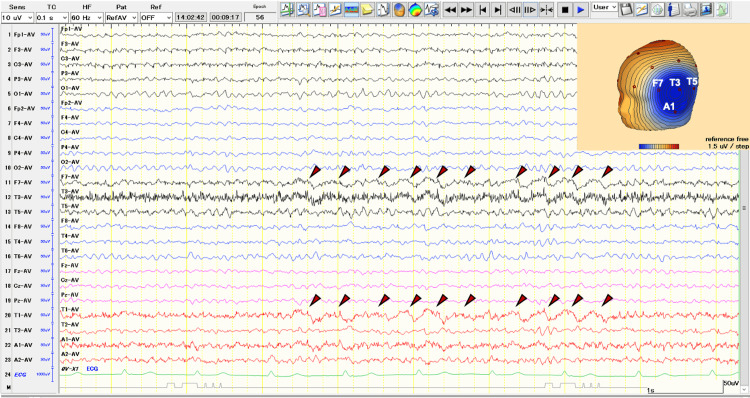
Interictal EEG EEG on the average montage shows semi-rhythmic delta waves (2-3 Hz) on the left frontotemporal region (red dotted allow line).

On examination, he exhibited no neurological abnormal findings. Brain MRI showed no abnormalities, yet EEG showed intermittent semi-rhythmic delta activity in the left temporal region (Figure 1).

**Figure 2:**
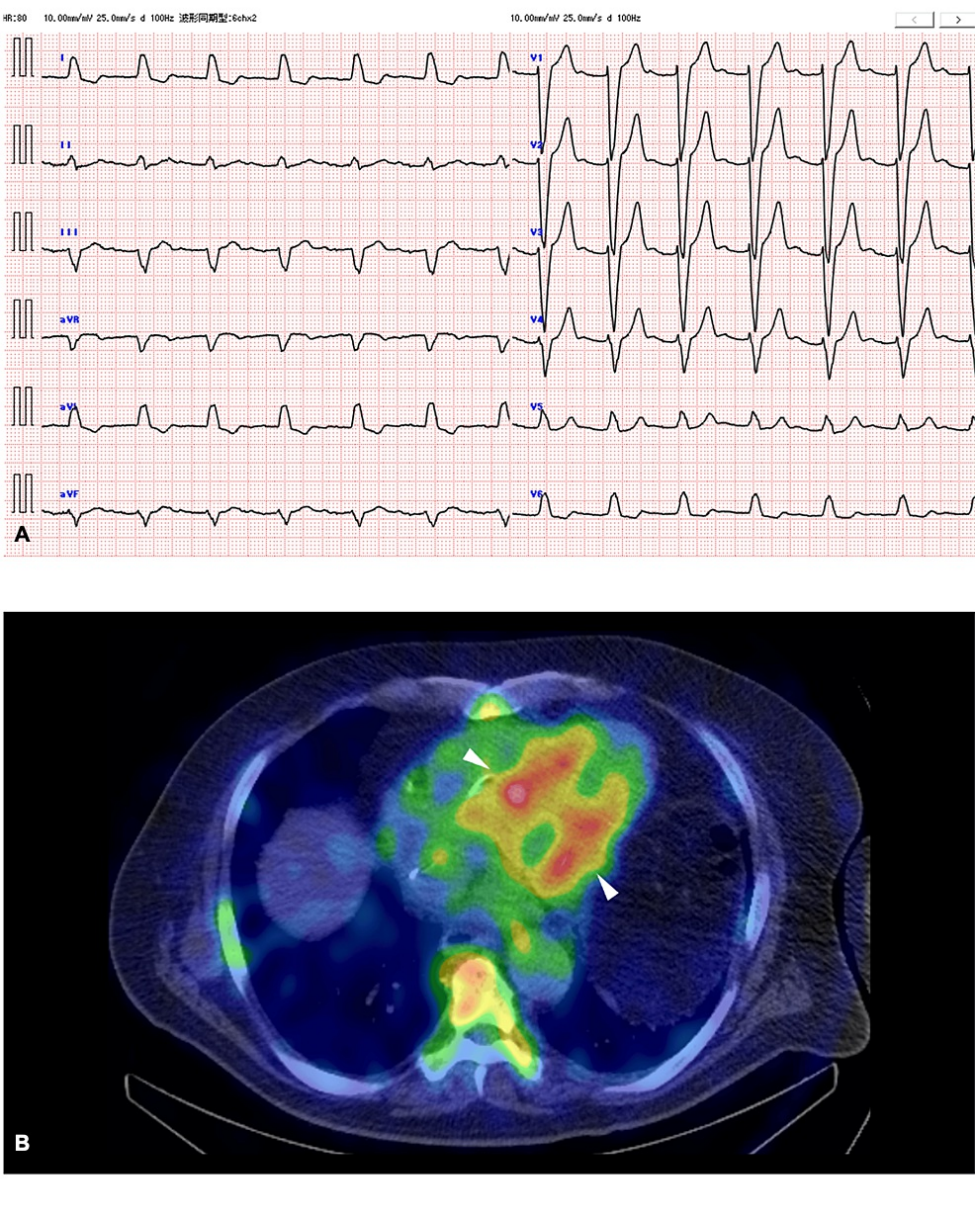
ECG and nuclear scintigraphy (A) ECG shows a left bundle branch block. (B) 99m-Technetium pyrophosphate scintigraphy illustrates myocardial uptake (white arrow head), suggesting a diagnosis of transthyretin amyloidosis.

These findings may suggest a possibility of a diagnosis of temporal lobe epilepsy with focal impaired awareness seizure. Conversely, ECG revealed a left bundle branch block (Figure 2A). Thus, Holter ECG was examined. Although the initial holter ECG for 24 hours shows unremarkable findings, subsequent monitoring for several days revealed a complete atrioventricular block (CAVB), necessitating permanent pacemaker implantation (during the event of CAVB, he reported abdominal discomfort almost identical to the previous one). Following this procedure, the episodes of loss of awareness ceased, and the abdominal aura was no longer observed. As 99m-Technetium pyrophosphate scintigraphy illustrates myocardial uptake (Figure 2B), a possible diagnosis of transthyretin amyloidosis was established.

## Discussion

The patient's initial report of epigastric discomfort raised suspicion of epileptic abdominal aura. However, upon thorough evaluation, it became evident that the symptoms were attributable to cardiogenic pre-syncope. It is noteworthy that pre-syncope episodes can manifest with a range of symptoms related to the autonomic nervous system [4]. Given that temporal lobe epilepsy may also present with autonomic manifestations, it is crucial to differentiate between these two conditions. An important distinguishing factor is the duration of the episodes. Epileptic events typically extend from 30 seconds to a few minutes [5], while pre-syncope manifestations are usually of shorter duration and are more commonly associated with physical exertion. Thus, the patient's presentation initially prompted consideration of an epileptic seizure.

Another critical distinguishing feature in this case was the presence of urinary incontinence in the absence of convulsion. Typically, epileptic seizures accompanied by incontinence occur within the context of a generalized seizure. In contrast, focal impaired awareness seizures in temporal lobe epilepsy occasionally exhibit urinary urge but rarely involve flaccid urinary incontinence [6]. Additionally, urinary incontinence often appears following convulsions or some kind of ictal motor manifestation in epilepsy [7]. Thus, we consider that a seizure without motor manifestations, including FIAS, may not easily exhibit urinary incontinence. Therefore, in this instance, the prevailing interpretation suggests that the patient experienced flaccid urinary incontinence due to syncope.

While it remains theoretically possible that cardiac arrest could occur during epileptic seizures associated with temporal lobe epilepsy (ictal asystole) [8], it is noteworthy that all events in the present case were resolved through pacemaker implantation without the need for anti-seizure medication. Therefore, it is imperative to underscore the importance of avoiding overdiagnosis of epilepsy solely on the grounds of unspecific EEG abnormalities such as focal slows. Additionally, urinary incontinence without convulsions might be a red flag for cardiogenic syncope rather than an epileptic seizure. Conversely, it is important not to disregard the EEG findings, and the possibility of occurrence of ictal asystole or ictal cardiac arrhythmia and the possibility of ictal nature of cardiac findings are not ruled out. Video EEG monitoring and capturing the events under question to make a claim to one over another accurately is essential (excluding one does not constitute a definitive diagnosis of the other).

## Conclusions

Based on our patient's history, differentiating between syncope and epileptic seizures posed a challenge. Initially, EEG findings suggested epilepsy due to the presence of slow waves in the temporal region. However, the occurrence of 'urinary incontinence without seizures' raised concerns (as this is not typical for a focal impaired awareness seizure) and prompted the need for continuous electrocardiogram monitoring. It is advisable to exercise caution when interpreting 'urinary incontinence without seizures' to avoid over-interpretation of EEG results.
